# Unveiling a rare haemorrhagic malignant pleural effusion: The role of medical thoracoscopy in diagnosing primary pleural angiosarcoma

**DOI:** 10.1002/rcr2.70068

**Published:** 2024-11-25

**Authors:** Ad Rian Chong, Khai Lip Ng, Nai‐Chien Huan, Nur Husna Mohd Aminudin, Maryam Ahmad Sharifuddin, Raja Nor Adilla Raja Rahaizat, Kasuma Mohamed Nordin

**Affiliations:** ^1^ Division of Respiratory Medicine, Department of Internal Medicine Melaka Hospital Melaka Malaysia; ^2^ Department of Respiratory Medicine Queen Elizabeth Hospital Kota Kinabalu Malaysia; ^3^ Department of Pathology Melaka Hospital Melaka Malaysia; ^4^ Department of Radiology Melaka Hospital Melaka Malaysia

**Keywords:** angiosarcoma, medical thoracoscopy, pleural effusion, primary pleural angiosarcoma

## Abstract

Primary pleural angiosarcoma (PPA) is a rare and challenging tumour to diagnose, often mistaken for other malignancies such as mesothelioma and lung cancer due to overlapping clinical and imaging features. We report a 52‐year‐old woman who presented with progressive shortness of breath and pleuritic chest pain. Imaging studies and thoracentesis revealed a large haemorrhagic left pleural effusion. Medical thoracoscopy (MT) showed a thickened and lobulated parietal pleura with multiple nodular lesions. Histopathological examination confirmed a diagnosis of angiosarcoma, characterized by pleomorphic tumour cells, a high Ki67 proliferation index and positive immunohistochemical markers, including CD31, D2‐40, Vimentin, and Factor VIII. Tragically, the patient developed a hospital‐acquired infection and passed away before any definitive treatment for the angiosarcoma could be initiated. This case underscores the diagnostic complexities of PPA and highlights the utility of MT in identifying this rare malignancy.

## Graphical abstract

Our case report details the diagnosis of primary pleural angiosarcoma (PPA) using medical thoracoscopy (MT), highlighting the procedure's key role in identifying rare malignancies.

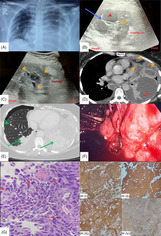

## INTRODUCTION

Angiosarcoma is a rare form of sarcoma originating from the endothelial cells, accounting for only 1%–2% of all soft tissue sarcomas.^1^ While angiosarcoma can develop in various body sites, it most commonly affects cutaneous tissues, particularly in the head and neck region, as well as soft tissues, visceral organs, bones, and the retroperitoneum.^2^ Primary pleural angiosarcoma (PPA) is especially rare and is known for its aggressive behaviour and poor prognosis.^3^, ^4^ Diagnosis is challenging due to clinical and radiological similarities to mesothelioma, pulmonary carcinoma, and metastatic adenocarcinoma.^5^ Our case report details the diagnosis of PPA using medical thoracoscopy (MT), highlighting the procedure's key role in identifying rare malignancies.

## CASE REPORT

**FIGURE 1:**
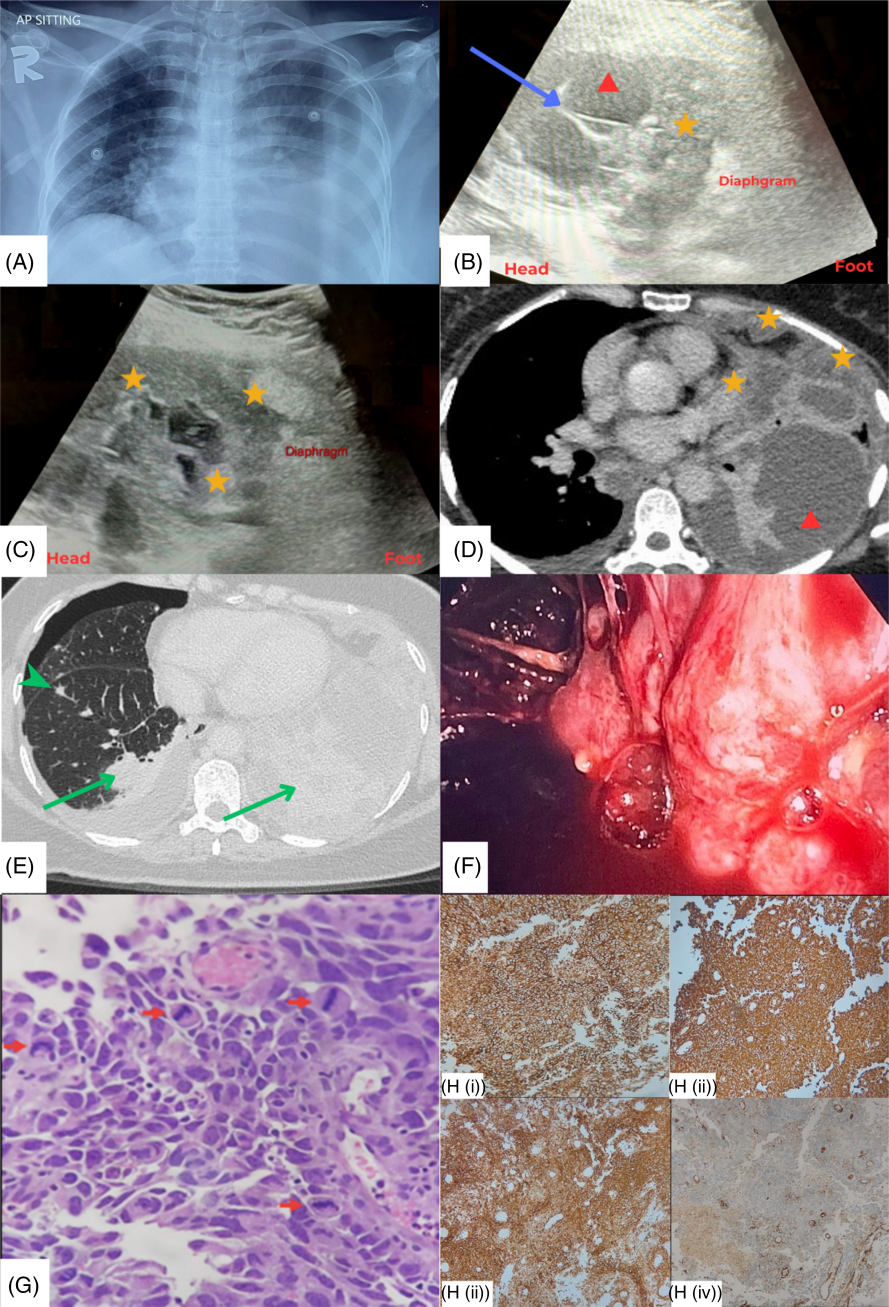
(A) Chest radiograph showed moderate loculated left pleural effusion. (B) Longitudinal ultrasound of the left hemithorax showed thickened pleura with multiple septations (blue arrow), pleural fluid (red triangle) with lobulated hypoechoic pleural mass (yellow star). (C) Longitudinal ultrasound of the left hemithorax showed lobulated hypoechoic pleural mass abutting the diaphragm (yellow star). (D) Computed tomography (CT) of the thorax showed lobulated heterogeneously enhancing costal and mediastinal pleural mass (yellow star), with loculated hypodense left pleural effusion (red triangle). (E) CT of the thorax showed ill‐defined hypodense nodules in both lower lobes (green arrow), with a solid nodule with spiculated margin in the right hemithorax (green arrowhead). Mild pleural effusion and pneumothorax were observed in the right hemithorax. (F) Medical thoracoscopy revealed thickened, irregular and lobulated parietal pleura with dense adhesions. (G) The tumour cells are arranged in sheets and papillary architecture with a fibrovascular core, which exhibits marked pleomorphism, hyperchromatic nuclei, and minimal eosinophilic cytoplasm. Mitosis is frequent (red arrow) (haematoxylin‐eosin, × 400). (H) The tumour cells were immunoreactive for CD31 (Hi), D2‐40 (Hii), Vimentin (Hiii), and factor VIII (Hiv) (×200).

A 52‐year‐old woman with a history of multinodular goitre presented to the casualty with a two‐week history of shortness of breath and left‐sided pleuritic chest pain. She had no constitutional symptoms, cough, or haemoptysis. Physical examination showed reduced breath sounds and stony dullness on percussion of the left hemithorax. There were no notable skin lesions. Chest radiography revealed a left pleural effusion (Figure 1A). Bedside thoracic ultrasound showed a left pleural effusion with a lobulated hypoechoic pleural mass abutting the diaphragm (Figure 1B,C). Blood investigations were as below: haemoglobin 11.4 g/L, white blood count 13.5 × 10^3^/μL, platelet 448 × 10^9^/L, urea 4.3 mmol/L, sodium 142 mmol/L, potassium 4.1 mmol/L, chloride 106 mmol/L, creatinine 64 μmol/L, total protein 80 g/L, total bilirubin 6.3 μmol/L, albumin 43 g/L, globulin 37 g/L, alanine transaminase (ALT) 12 U/L, alkaline phosphatase (ALP) 96 U/L, aspartate aminotransferase (AST) 19 U/L, and C‐reactive protein (CRP) 60 mg/L. She was initially treated for pneumonia with parapneumonic effusion due to elevated inflammatory markers.

A pigtail catheter was inserted, and the pleural fluid was haemorrhagic. The effusion was exudative with pleural fluid lactate dehydrogenase (LDH) levels of 1139 U/L, and pleural fluid protein levels of 56 g/L. Pleural fluid cytology cell block revealed atypical cells, while the pleural fluid adenosine deaminase level was elevated at 45.04 U/L (normal regional cutoff <29.6). Tuberculosis cultures, along with bacterial cultures, were negative. A computed tomography (CT) scan of the thorax, abdomen, and pelvis revealed lobulated, heterogeneously enhancing masses in the costal and mediastinal pleura, accompanied by a loculated hypodense left pleural effusion. Multiple lung nodules were noted in both lung fields, the largest at the left lower lobe, measuring 2.9 × 2.6 × 2.4 cm, and enlarged mediastinal lymph nodes consistent with metastasis (Figure 1D,E). No masses were identified in the abdomen or pelvis. An incidental finding of a right hydropneumothorax was also noted (Figure 1E). As video‐assisted thoracoscopy (VATS) and surgical biopsy were not available in our centre, a decision was made to proceed with MT, which was performed 1 week after admission.

During MT, which was performed under conscious sedation, biopsy forceps was used to dissipate pleural septations. A thickened and lobulated parietal pleura lined with multiple nodules and masses were observed post‐mechanical adhesiolysis (Figure 1F). A total of 10 pleural biopsies were taken from the mid‐thoracic posterior parietal pleura for microbiological and histopathological studies. A chest drain was left for 2 days post‐MT, and a total of 1.5 L of haemorrhagic pleural fluid was drained.

Histopathological analysis of the parietal pleura showed a tumour organized in sheets and papillary structures with a fibrovascular core. The tumour cells exhibit marked pleomorphism and hyperchromatic nuclei, with minimal eosinophilic cytoplasm (Figure 1G). Immunohistochemical staining shows that the tumour cells were positive for CD31, D2‐40, Vimentin, and Factor VIII (Figure 1H), while negative for CKAE1/AE3, CK7, CK20, EMA, TTF‐1, Napsin A, Synaptophysin, Chromogranin, CD56, HMB45, PAX8, WT1, CD34, P63, P40, CK5/6, ER, Calretinin, and Desmin. The Ki67 proliferation index was greater than 80%, consistent with a high‐grade neoplasm exhibiting vascular differentiation, confirming a diagnosis of angiosarcoma.

While awaiting the histopathological analysis, she developed septic shock, evidenced by temperature spikes, an elevated white blood cell count of 20.5 × 10^9^/L, and a raised CRP of 300.3 mg/L, along with a bloodstream infection from an extended‐spectrum beta‐lactamase (ESBL)‐producing *Klebsiella pneumoniae*. She was initially started on empirical piperacillin‐tazobactam and was subsequently switched to targeted antibiotic therapy with meropenem. An oncology consultation was conducted, but the patient was deemed unfit for cancer‐specific therapy as she was critically ill. Unfortunately, her oxygen requirements escalated, necessitating intubation and increased inotropic support. This progression ultimately led to multiorgan failure and eventually, her demise.

## DISCUSSION

Most lung and pleural angiosarcomas are metastatic; primary cases are exceptionally rare and often diagnosed late due to a low index of suspicion.^4^ Pleural fluid cytology yields an overall diagnostic rate of 60% for malignancy, often requiring further testing. In our patient, the haemorrhagic effusion showed only atypical cells on cytology.^6^ Blind pleural biopsies generally have a low diagnostic yield for malignancy.^4^, ^5^, ^6^, ^7^ While VATS or surgical biopsy is more effective, these methods may not always be accessible. At our centre, where VATS and surgical biopsy are unavailable, MT has proven to be a valuable alternative, enabling successful diagnosis. Unlike VATS, MT can be performed under sedation with local anaesthesia, making it a cost‐effective choice, especially for frail patients with multiple comorbidities. MT requires only a single incision for trocar placement, resulting in less tissue trauma, reduced postoperative pain, and quicker recovery. It also has a favourable safety profile, with major complications occurring in just 1.8% of cases, according to Giona et al.'s review of over 4000 MT cases. While extensive pleural adhesions can increase the risk of lung laceration during trocar insertion, no significant bleeding has been reported in the literature, likely due to direct tumour visualization during MT or surgical biopsy.^7^

Patients with PPA can present with symptoms such as pleuritic chest pain, shortness of breath, haemoptysis, cough, weight loss and recurrent haemothorax.^2^ Radiologic evaluation typically reveals unilateral or bilateral pleural effusion and focal or diffuse pleural thickening, mimicking mesothelioma. Sedhai et al. reported that mass‐like lesions from the lung are observed in about half of the cases of PPA, and there are no distinct radiologic characteristics to differentiate PPA from other metastatic and primary pleural tumours. Commonly reported metastatic sites are lymph nodes, bone, brain, liver, spleen, adrenals, skin, oral cavity, and spinal cord.^4^, ^5^ Chang et al. conducted a literature review and identified 24 patients with PPA who presented with spontaneous hydropneumothorax. When PPA invades the lungs, pneumothorax or haemothorax may occur due to the formation of nodular lesions or cystic changes, as seen in our patient, who developed a spontaneous right hydropneumothorax secondary to metastases.^8^ Predisposing factors for angiosarcoma include exposure to polyvinyl chloride and thorium dioxide, postmastectomy and post‐irradiation states, and chronic empyema.^9^ Reports from Japan have associated PPA with tuberculous pyothorax, while Western cases have linked it to asbestos exposure and radiotherapy. The average duration of chronic tuberculous pyothorax before the onset of angiosarcoma is approximately 30 years, indicating the need for long‐term observation.^10^, ^11^ Our patient did not have any of the risk factors, suggesting a de novo origin of her PPA.

Treatment modalities for PPA include surgery, radiotherapy, and chemotherapy. Surgery followed by adjuvant radiotherapy is considered the optimal treatment approach, although there are no consensus criteria for chemotherapy regimens.^12^ Chemotherapy drugs used include gemcitabine, docetaxel, paclitaxel, cisplatin, ifosfamide, doxorubicin, and albumin‐bound paclitaxel.^13^ Pazopanib, a tyrosine kinase inhibitor, is an emerging targeted therapy that has demonstrated superiority in median progression‐free survival and overall survival when used in phase 3‐trial patients with PPA.^14^ Additionally, immune checkpoint inhibitors such as pembrolizumab and nivolumab plus ipilimumab have also shown success in treating angiosarcoma.^15^ The prognosis for PPA is poor, with a median overall survival of only four months. Approximately 80% of patients died from PPA within 10 months of diagnosis, and the 2‐year survival rate was approximately 4.4%.^16^ Factors linked to a worse prognosis include the presence of pleural effusion, haemothorax, tumour size greater than 5 cm, and high‐grade histological differentiation. The presence of haemothorax was an independent predictor of worse prognosis.^2^, ^16^ This highlights the need for further research into the etiopathogenesis and optimal treatment strategies for this rare malignancy.

In conclusion, this case underscores the critical role of comprehensive diagnostic evaluation, including advanced imaging and medical thoracoscopy, in identifying rare pleural malignancies. Clinicians should maintain a high index of suspicion for PPA in patients with unexplained haemorrhagic pleural effusions and atypical cytological findings to facilitate timely diagnosis and potential treatment. Further research is needed to better understand the etiopathogenesis and optimal treatment strategies for this rare and aggressive malignancy.

## AUTHOR CONTRIBUTIONS

Ad Rian Chong, Khai Lip Ng, Nai‐Chien Huan contributed to the design and implementation of the case report. Ad Rian Chong, Khai Lip Ng, Nai‐Chien Huan wrote the manuscript. Khai Lip Ng and Nur Husna Mohd Aminudin carried out the procedure and treatment mentioned. Maryam Ahmad Sharifuddin interpreted the pathological slides. Raja Nor Adilla Raja Rahaizat reviewed all the radiological images and interpreted the radiological findings. Kasuma Mohamed Nordin supervised the project. All authors discussed the study and contributed to the final manuscript.

## CONFLICT OF INTEREST STATEMENT

None declared.

## ETHICS STATEMENT

The authors declare that appropriate written informed consent was obtained for the publication of this manuscript and accompanying images.

## Data Availability

Data sharing is not applicable to this article as no new data were created or analyzed in this study.
